# A Novel Case of Sarcoidosis‐Lymphoma Syndrome

**DOI:** 10.1155/crgm/8829729

**Published:** 2026-08-25

**Authors:** Mario Tavakoli, Alexander Pan, Sam Papasotiriou

**Affiliations:** ^1^ Department of Medicine, University of Illinois Hospital, Chicago, Illinois, USA, duhs.edu.pk; ^2^ Digestive Health Institute, Northwestern Medicine, Chicago, Illinois, USA, nm.org

**Keywords:** gastric sarcoidosis, MALT lymphoma, sarcoidosis-lymphoma syndrome

## Abstract

Sarcoidosis‐lymphoma syndrome (SLS) is a rare condition that elucidates the association between sarcoidosis and the development of lymphoma. It is believed that the chronic active inflammation from sarcoidosis increases the risk of malignant transformation of lymphoid cells. There are only 4 documented cases of mucosa‐associated lymphoid tissue (MALT) lymphoma in patients with sarcoidosis, and we present the first reported case of a patient with *Helicobacter pylori* (*H. pylori*)‐negative gastric MALT lymphoma preceding the diagnosis of gastric sarcoidosis.

## 1. Introduction

First described by Brincker in 1989, sarcoidosis‐lymphoma syndrome (SLS) is a rare condition that elucidates the association between sarcoidosis and the development of lymphoma [1]. Although the correlation between the two pathologies remains controversial, it is believed that the chronic inflammatory status from sarcoidosis increases the risk of malignant transformation of lymphoid cells via dysregulation of immunologic cell patterns [2]. In conventional cases of SLS, patients develop a lymphoma approximately one to 2 years after the onset of sarcoidosis. A small subset of patients with a history of leukemia or lymphoma goes on to develop late‐onset sarcoidosis, and the pathophysiology of this presentation remains uncertain [3]. Although there are approximately 79 reported cases of SLS, there is no clear pattern for how this syndrome manifests itself [4]. In the literature, sarcoidosis has been classically associated with Hodgkin’s lymphoma, although it has also been linked to the development of non‐Hodgkin lymphomas such as follicular lymphoma, diffuse large B‐cell lymphoma, orbital lymphoma, and mucosa‐associated lymphoid tissue (MALT) lymphoma [4]. There are only four documented cases of MALT lymphoma in patients with sarcoidosis, and we present the first reported case of a patient with *Helicobacter pylori* (*H. pylori*)‐negative gastric MALT lymphoma preceding the diagnosis of gastric sarcoidosis.

## 2. Case Presentation

A 51‐year‐old African American man presented to the Gastroenterology clinic for evaluation of early satiety and unintentional weight loss. The patient endorsed experiencing a cough with episodes of posttussive emesis and shortness of breath, although he denied any abdominal pain, nausea, vomiting, reflux, or dysphagia. His past medical history included obstructive sleep apnea, hyperlipidemia, meningitis, and diet‐controlled irritable bowel syndrome with diarrhea predominance. Initial laboratory results were notable for hemoglobin (12.7 g/dL), aspartate aminotransferase (30 U/L), alanine transaminase (68 U/L), alkaline phosphatase (180 U/L), total bilirubin (0.7 mg/dL), and angiotensin‐converting enzyme (ACE) level (83 U/L). A computed tomography (CT) scan of the lungs revealed hilar lymphadenopathy, suggesting that an endobronchial ultrasound‐guided transbronchial needle aspiration (EBUS‐NA) should be performed. Although the biopsies were negative for high‐grade malignant cells or acid‐fast bacteria (AFB) staining, they revealed changes that may represent a granulomatous lesion.

**FIGURE 1:**
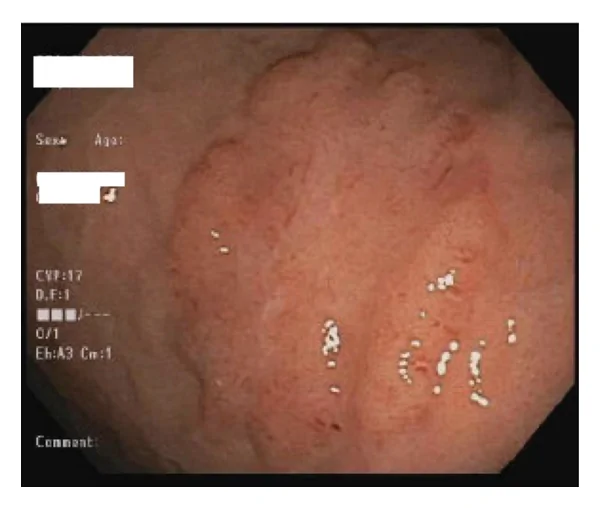
EGD revealing a single 30–35‐mm flat lesion in the greater curvature of the gastric body. Biopsy staining revealed MALT lymphoma with massive B‐cell infiltration of the gastric mucosa.

The patient then underwent esophagogastroduodenoscopy (EGD) due to continued weight loss and early satiety, which revealed esophagitis and a single 30–35‐mm flat lesion with no stigmata of bleeding in the greater curvature of the gastric body (Figure 1). Although the biopsy results were negative for *H. pylori*, they revealed massive B‐cell infiltration of the gastric mucosa, with staining being indicative of MALT lymphoma.

The patient was simultaneously diagnosed with pulmonary sarcoidosis and completed a prednisone taper, which significantly improved his cough and dyspnea. A repeat surveillance EGD was performed, which revealed a 30–40‐mm flat lesion on the greater curvature, along with a 15‐mm flat lesion with irregular mucosa on the proximal antrum. The morphologies of the two lesions were similar to those of the previous biopsies and were negative for *H. pylori*.

**FIGURE 2:**
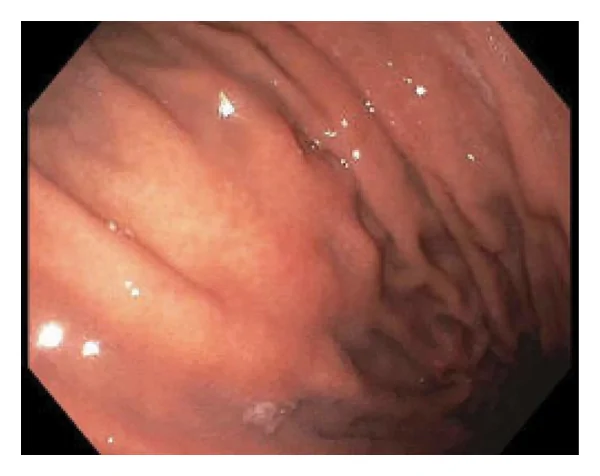
Final EGD depicting scattered areas of mild localized erythema in the gastric body. A diagnosis of gastric sarcoidosis was made, as biopsies revealed noncaseating granulomas with chronic inflammatory cells composed of cluster of differentiation (CD) 20 and CD79 positive B‐cells, along with CD3 and CD5 positive T‐cells.

**FIGURE 3:**
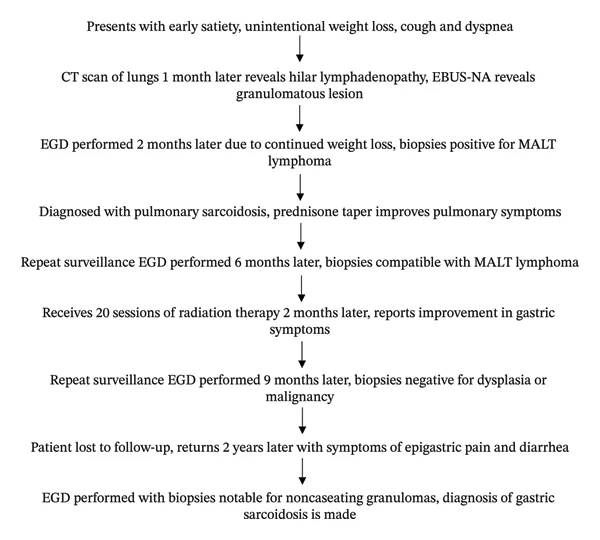
Timeline of events in chronological order with time elapsed between each diagnostic step.

The patient was then referred to the Radiation Oncology clinic and received 20 sessions of radiation therapy, after which he felt like he lost sensation in his stomach and no longer felt hungry or full. A repeat surveillance EGD 9 months following radiation revealed congested and erythematous mucosa in the antrum and prepyloric regions. Biopsies at that time were negative for *H. pylori*, intestinal metaplasia, dysplasia, or malignancy. Although a repeat EGD was scheduled for 6 months, the patient was lost to follow‐up and returned to the Gastroenterology clinic 2 years later, complaining of epigastric pain and diarrhea after eating a red pepper. An EGD at that time revealed esophagitis with scattered areas of mild localized erythema in the gastric antrum and body (Figure 2). Although the biopsy results were negative for fungal and AFB stains, they revealed noncaseating granulomas with chronic inflammatory cells, and a diagnosis of gastric sarcoidosis was made. Biopsies at this time were also negative for *H. pylori*. We have included a timeline of events in chronological order with the time elapsed between each diagnostic step (Figure 3).

## 3. Discussion

While the coexistence of sarcoidosis and lymphoma has been reported previously, the case we are describing is unique in several respects. In the literature, it is estimated that patients with sarcoidosis are up to 11 times more likely to develop lymphoma than the general population [1]. However, less than 20% of reported cases describe a nonconventional form of SLS where the malignancy develops first, as in the case we have described previously [4]. Although there have been four documented cases of patients with sarcoidosis and MALT lymphoma, two of the cases described patients with pulmonary lymphoma [5, 6]. The other two cases reported patients with gastric MALT lymphoma, although they were both positive for *H. pylori* [7, 8]. Infection with *H. pylori* significantly increases the risk of developing gastric MALT lymphoma, which makes the case we have described particularly novel. Our patient appears to be the first documented case of *H. pylori*‐negative gastric MALT lymphoma accompanying a diagnosis of sarcoidosis in the literature.

The earliest reports of SLS were described by Brincker, who examined cases of sarcoidosis in patients who presented with malignant tumors in the Danish Cancer and Sarcoidosis Registries [9]. Further studies have attempted to quantify the incidence of sarcoidosis and non‐Hodgkin lymphomas, with one study showing a notable increase in the risk of lymphoma in patients with a previous history of sarcoidosis (odds ratio: 1.9) [10]. This observed association may be due to significant changes in the immune systems of patients with autoimmune diseases, such as sarcoidosis. Noor and Knox reported that the increased mitogenesis and hyperstimulation of T and B lymphocytes observed in sarcoidosis likely predisposes patients to the development of malignancies in the lymphoid system [3]. The exact cause of the pathogenesis of nonconventional SLS is still unclear. It is believed that chemotherapy administered to patients with lymphoma contributes to worsening clinical manifestations of underlying sarcoidosis [4]. The patient presented in our case report was not treated with chemotherapy, which makes this case particularly unusual.

The diagnosis of gastric sarcoidosis in this patient is somewhat obscured for a number of reasons. The patient received radiation therapy which is associated with the development of granulomas, and granuloma formation following lymphoma treatment is a recognized phenomenon which can mimic a sarcoid‐like reaction [11]. Foreign material including medications and retained vegetable matter can also manifest as noncaseating granulomas, and the pathology report did not mention any absence of foreign bodies. However, a prior diagnosis of pulmonary sarcoidosis with granulomatous changes seen on EBUS supports an additional diagnosis of gastric manifestations of sarcoidosis over another process. Given the patient’s history of IBS‐D, there was no prior evidence of Crohn’s disease seen on previous colonoscopies which could also present with gastric granulomas. While this case report was written at the time of diagnosis of gastric sarcoidosis, it would be interesting to note if the patient responded well to sarcoidosis treatment, if the granulomas persisted or if the lymphomas reoccurred. Persistence of granulomas following sarcoidosis treatment would support another etiology over a diagnosis of gastric sarcoidosis. Biological agents and corticosteroids prescribed in patients with sarcoidosis can increase the susceptibility of developing lymphoproliferative malignancies such as MALT lymphoma [12]. This highlights the importance of attentive monitoring of lymphoma in patients with sarcoidosis, as this disease can often imitate and promote the development of lymphoma [12]. In conclusion, this case highlights the likely association between non‐Hodgkin lymphoma and sarcoidosis, while describing a novel presentation of SLS.

**Nomenclature**
AFB: Acid‐fast bacteria
ACE: Angiotensin‐converting enzyme
CD: Cluster of differentiation
CT: Computed tomography
EBUS‐NA: Endobronchial ultrasound‐guided transbronchial needle aspiration
EGD: Esophagogastroduodenoscopy
*H. pylori*
: *Helicobacter pylori*
MALT: Mucosa‐associated lymphoid tissue lymphoma
SLS: Sarcoidosis‐lymphoma syndrome

## Author Contributions

Mario Tavakoli completed the Introduction, Discussion, and final revisions. Sam Papasotiriou described the case presentation. Alexander Pan saw the case and contributed by providing insight and helped with the final review.

## Funding

This manuscript was not funded.

## Disclosure

All authors have read the manuscript and approved its submission.

## Ethics Statement

This retrospective case report was considered exempt from the University of Illinois Chicago Institutional Review Board oversight.

## Conflicts of Interest

The authors declare no conflicts of interest.

## Data Availability

Data sharing is not applicable to this article as no datasets were generated or analyzed during the current study.

## References

[1] Brincker H. , The sarcoidosis-lymphoma Syndrome, British Journal of Cancer. (1986) 54, no. 3, 467–473, 10.1038/bjc.1986.199. 3756082 PMC2001616

[2] Cohen P. R. and Kurzrock R. , Sarcoidosis and Malignancy, Clinics in Dermatology. (2007) 25, no. 3, 326–333, 10.1016/j.clindermatol.2007.03.010. 17560310

[3] Noor A. and Knox K. S. , Immunopathogenesis of Sarcoidosis, Clinics in Dermatology. (2007) 25, no. 3, 250–258, 10.1016/j.clindermatol.2007.03.002. 17560302

[4] Goswami T. , Siddique S. , Cohen P. , and Cheson B. D. , The Sarcoid-Lymphoma Syndrome, Clinical Lymphoma, Myeloma & Leukemia. (2010) 10, no. 4, 241–247, 10.3816/clml.2010.n.052. 20709659

[5] Baig N. , Rao R. D. , Daniel V. , Kandaswamy C. , and Vedire A. , Association of Pulmonary MALT Lymphoma and Sarcoidosis, Blood. (2020) 136, no. S1, 19–20, 10.1182/blood-2020-140071.

[6] Kokuho N. , Terasaki Y. , Urushiyama H. et al., Pulmonary mucosa-associated Lymphoid Tissue Lymphoma Associated with Pulmonary Sarcoidosis: a Case Report and Literature Review, Human Pathology. (2016) 51, 57–63, 10.1016/j.humpath.2015.12.019. 27067783

[7] Fukuda T. , Sato K. , Tachikawa S. , Ohnuki K. , Ohtani H. , and Suzuki T. , Mucosa‐Associated Lymphoid Tissue Lymphoma Coexisting with Epithelioid Granulomas in the Stomach of a Patient with Systemic Sarcoidosis, Pathology International. (1997) 47, no. 12, 870–875, 10.1111/j.1440-1827.1997.tb03720.x. 9503470

[8] Torchio M. , Bottaro G. , Bertolino G. et al., Late-Onset Sarcoidosis in a Patient with Gastric mucosa-associated Lymphoid Tissue non-Hodgkin Lymphoma: a Case Report, Oncology Letters. (2014) 8, no. 3, 1299–1301, 10.3892/ol.2014.2241. 25120711 PMC4114627

[9] Brincker H. and Wilbek E. , The Incidence of Malignant Tumours in Patients with Respiratory Sarcoidosis, British Journal of Cancer. (1974) 29, no. 3, 247–251, 10.1038/bjc.1974.64. 4830144 PMC2009094

[10] Mellemkjaer L. , Pfeiffer R. M. , Engels E. A. et al., Autoimmune Disease in Individuals and Close Family Members and Susceptibility to Non-hodgkin’s Lymphoma, Arthritis & Rheumatism. (2008) 58, no. 3, 657–666, 10.1002/art.23267. 18311836

[11] Du J. , Zhang Y. , Liu D. , Zhu G. , and Zhang Q. , Hodgkin’s Lymphoma with Marked Granulomatous Reaction: a Diagnosis Pitfall, International Journal of Clinical and Experimental Pathology. (2019) 12, no. 7, 2772–2774. 31934112 PMC6949579

[12] Voutidou S. , Eleftheriadis D. , Drakopanagiotakis F. , Papanikolaou I. C. , and Steiropoulos P. , Pathogenic Mechanisms Linking Sarcoidosis to Lymphoma, International Journal of Molecular Sciences. (2025) 26, no. 2, 10.3390/ijms26020594. PMC11765988 39859309

